# Measuring the Efficiency of Fiscal Policies for Environmental Pollution Control and the Spatial Effect of Fiscal Decentralization in China

**DOI:** 10.3390/ijerph17238974

**Published:** 2020-12-02

**Authors:** Caihua Zhou, Xinmin Zhang

**Affiliations:** 1 School of Public Finance and Taxation, Zhejiang University of Finance and Economics, Hangzhou 310018, China; audrey0515@163.com; 2 Institute of Ecological Civilization, Jiangxi University of Finance and Economics, Nanchang 330013, China

**Keywords:** fiscal policies, environmental pollution control, technical efficiency, fiscal decentralization, spatial effect

## Abstract

This paper uses both fiscal expenditure policy and fiscal revenue policy as input indicators and selects environmental pollution control results reflecting different forms and sources of pollution as output indicators. The efficiency of fiscal policies for environmental pollution control (EFPE) of 30 provincial-level administrative divisions in China from 2007 to 2017 is measured by adopting the data envelopment analysis (DEA) method. Then, the spatial effect of fiscal decentralization on EFPE is empirically analyzed by using the spatial lag model (SLM). The results show that EFPE values in China have been greatly improved overall since 2014. The change in technical efficiency (TE) is caused mainly by the change in pure technical efficiency (PTE). EFPE values have regional heterogeneity and convergence. The eastern region has clearly higher EFPE values than other regions. The growth rate of the low efficient region is greater than that of the high efficient region. Fiscal expenditure decentralization has a direct negative effect and spatial spillover effect on EFPE values, while fiscal revenue decentralization has a non-significant effect. Based on these results, this paper proposes the following policy implications: increasing the level of fiscal expenditure of environmental pollution control and improving the central transfer payment system for environmental protection; reforming the government performance assessment system and innovating the conditions of government expenditure on environmental pollution control; and promoting horizontal fiscal cooperation in cross-regional environmental governance.

## 1. Introduction

Performance evaluation was first applied to government management in the performance budget system of the United States in the 1950s. Since the 1970s, “new public management” (NPM) [1] has been widely carried out in western countries. The NPM movement has led to performance evaluations being widely used in government management. An important part of government performance evaluation is evaluating the efficiency of the fiscal policies implemented by the government. Neo-public finance (NPF) [2], proposed by Chinese scholar Junsheng Li, has innovated the theoretical basis of government intervention in China. NPF shows that government intervention cannot completely solve the problem of market failure. The government is also a behavioral subject in the market in essence. To establish a socialist market economic system in China, measuring the efficiency of the fiscal policies is also the content of the modern fiscal and taxation system [3].

Since implementing the construction of ecological civilization [4,5,6,7], the Chinese government has been accelerating the introduction of relevant policies including increasing the fiscal input on environment. Taking the fiscal expenditure of environmental protection as an example, the average annual growth rate of China from 2007 to 2017 was 19.57% [8]. It can be seen that the scale of expenditure on environmental protection has been increasing since 2007, accounting for a slight increasing proportion of gross domestic product (GDP) (Appendix A Table A1). The expenditure of local governments is the main source of whole country expenditure. The construction of ecological civilization faces a tough battle against environmental pollution, which includes air pollution, water pollution and soil pollution which have direct impacts on environmental quality and biological health. To promote the ecological civilization program by using fiscal policies, it is necessary to measure the efficiency of fiscal policies for environmental pollution control (EFPE) in China.

Fiscal federalism is manifested as fiscal decentralization in China. The Chinese government began market-oriented economic system reforms in 1978, and fiscal decentralization was an important aspect of the transition from a planned economy to a market economy [9]. After adjusting the fiscal relationship between the central and local governments through the tax distribution reform in 1994, China’s fiscal “top-down” decentralization has emphasized the mastery of resources and a leading role in macroeconomic regulation by the central government. Local governments are the representatives of the central government to a great extent. In regard to government environmental pollution control, fiscal decentralization results in environmental federalism [10]. Furthermore, due to the influence of local government competition and other factors, the impact of fiscal decentralization on environmental pollution control is relatively complex and needs to be further analyzed [11]. All provinces in China no longer meet the econometrics hypothesis of independence due to the imitation and comparison among provinces under the unified national policy and performance evaluation objectives. Therefore, it is more realistic to consider the spatial autocorrelation among provinces. Geopolitical analysis is proved to be important in relation to the efficiency of local governments [12]. In addition, considering the spillover of environmental pollution, geographical factors should be considered in the analysis of environmental pollution control [13].

Generally, effect analysis of fiscal policies on environmental pollution control is carried out before efficiency analysis. Fiscal policies of environmental pollution control can be divided into fiscal expenditure policy and fiscal revenue policy. Among them, fiscal expenditure policies of environmental pollution control are generally considered to have a negative effect on environmental pollution [14,15,16]. Some scholars have analyzed the pollution control effect of environmental fiscal expenditure according to the direct effect and indirect effect and found that the pollution control effect of fiscal input on different pollutants was different [17,18]. The “double dividend” theory of environmental taxation [19] shows that the fiscal revenue policy of environmental pollution control can not only improve environmental quality and obtain environmental dividends but also promote consumption and obtain efficiency dividends [20,21]. Some scholars have defined and developed a methodology for assessing and adapting to the effect and efficiency of environmental taxes [22,23] using the computable general equilibrium (CGE) model to simulate the effects of different Chinese environmental tax policies on the emissions of environmental pollutants. The effects of fiscal expenditure policies and revenue policies of environmental pollution control are also compared and found that they play different roles in different conditions [24,25].

With the establishment of the concept of fiscal expenditure efficiency in China [26], many scholars have carried out studies to measure the efficiency of fiscal policies for environmental pollution control based on effect analysis. The data envelopment analysis (DEA) method has been widely applied in efficiency evaluation, especially in public policy [27,28,29]. Scholars used the DEA method to measure the industrial pollution control expenditure of Chinese provincial governments [30,31,32] and the efficiency of fiscal policies for environmental pollution control by selecting both industrial pollution and domestic pollution as the output indicators [33,34]. Fare et al. [35] used the Malmquist index to calculate the total factor productivity change (TFPCH) based on the DEA method and Shephard’s distance functions and this approach has been widely applied in analyzing the change of efficiency value [36]. In addition, some scholars have transformed qualitative evaluation indicators into L-R fuzzy numbers for fuzzy DEA evaluation of policy performance [37] and adopted the analytic hierarchy process (AHP) [38,39], content analysis method [40] and stochastic frontier analysis (SFA) [41] to conduct performance evaluations of the fiscal policies for environmental pollution control.

The influencing factors of government efficiency are very diverse [42]. Among them, Chinese-style fiscal decentralization is believed to cause the extensive economic growth of local governments at the cost of high energy consumption and high pollution [43,44,45]. Under the unified national performance assessment system, competition among local governments will affect policy choices and thus affect the strength of fiscal policies for environmental pollution control [46,47,48]. The mismatch between financial power and administrative power also leads to a lack of local government enthusiasm and initiative in the process of environmental pollution control [49,50]. The existing studies have also shown that fiscal decentralization generally exacerbates environmental pollution [51,52] but has different effects on different spillover pollutants [53]. Han and Meng [50] studied the spatial effect of fiscal revenue decentralization and fiscal expenditure decentralization on the ecological environment. He [54] found that fiscal decentralization had no significant impact on environmental pollution but had a significant positive impact on fiscal expenditure of pollution control. In addition, Fu [55] believed that fiscal decentralization reduced the efficiency of the supply of non-economic public goods but did not reduce public input itself. The influence of fiscal decentralization on the environmental protection expenditure efficiency of local governments has been analyzed. The results show that there is a significant negative relationship between these factors [56,57], and different levels of fiscal decentralization have different impacts on environmental pollution control performance [58].

The above analyses show that the DEA method is frequently adopted to measure the efficiency of fiscal policies of environmental pollution control. However, existing research is incomplete in some areas. For example, only fiscal expenditure for environmental pollution control is selected as the input indicator [30,31,32]. Environmental pollution control for a certain source of pollution (e.g., industrial pollution) or a certain form of pollutant (e.g., waste gas pollution) is selected as an output indicator [33,34]. EFPE values are measured by using the DEA method in this paper. But what different from previous studies is that the fiscal expenditure policy and the fiscal revenue policy of environmental governance are used as input indicators, and the environmental pollution control results reflecting different pollution forms (wastewater, solid waste and waste gas) and different pollution sources (industrial pollution and domestic pollution) are selected as output indicators. The influences of fiscal decentralization on the implementation of environmental pollution control policy and the effect of policies for environmental pollution control have been mainly analyzed, but few studies have examined the influence of fiscal decentralization on the efficiency of fiscal policies for environmental pollution control [51,52,53]. There are many studies on the temporal effect but few on the spatial effect of fiscal decentralization [55,56,57,58]. In addition, most studies have not subdivided the indicators of fiscal decentralization and thus have been unable to reflect the differences [50]. To fill in the gaps in existing research, the spatial lag model (SLM) is used to empirically test the direct and spatial spillover effects of fiscal expenditure decentralization and fiscal revenue decentralization on EFPE in this paper.

On this basis, this paper proposes the question that needs to be solved urgently in China, that is, what is the efficiency of fiscal policies for environmental pollution control in China? Does fiscal decentralization affect the efficiency of fiscal policies for environmental pollution control? Is there a spatial effect of fiscal decentralization? What policies and suggestions could be proposed to improve the efficiency of fiscal policies for environmental pollution control in China? It innovates the indicators of input and output in the efficiency measurement of fiscal policies for environmental pollution control, considers the spatial effect in the analysis of influencing factors, and subdivides the fiscal decentralization into variables with Chinese characteristics. Therefore, this paper has theoretical and practical contributions under the background of accelerating the ecological civilization construction and establishing modern fiscal system in China. The structure of this paper is organized as follows. Section 2 introduces the DEA approach and data. Section 3 explores the efficiency measurement and convergence analysis. Section 4 analyzes the spatial effect of fiscal decentralization on EFPE. Finally, conclusions and suggestions are summarized in Section 5.

## 2. Methods and Data

### 2.1. DEA Approach

As a non-parametric estimation method, DEA can evaluate the efficiency of decision-making fairly and objectively, so it has been widely applied in social and economic fields. It first analyzes the input and output data of the decision-making unit (DMU). Then, the relative optimal value in the DMU is determined as the efficiency boundary. Thus, the comprehensive efficiency value of each DMU is calculated based on the gap between the DMU and the optimal DMU. This paper uses the DEA method to measure the efficiency of fiscal policies for environmental pollution control.

Suppose there are DMUs in n regions, i.e., $DMU_{j}\left(j=1,2,\cdots ,n\right)$. The fiscal policy input of environmental pollution control in each region is $X=\left(X_{1},X_{2},\cdots ,X_{n}\right)$. There are m kinds of outputs of fiscal policies for environmental pollution control (environmental pollution control results). The output vector of $DMU_{j}$ is $Y_{j}={\left(Y_{1j},Y_{2j},\cdots ,Y_{mj}\right)}^{T}$. Let $s^{-}$ and $s_{j}\left(j=1,2,\cdots ,m\right)$ be the slack variables of input and output, respectively, and ε be a non-infinitesimal of Archimedes. Then, the input-oriented CCR model [59] and BCC model [60] can be constructed.

The CCR model is constructed as Equation (1):

(1) $$\begin{array}{c} min\theta -\varepsilon \left(s^{-}+\overset{m}{\underset{j=1}{\sum }}s_{j}\right)=V_{D}\\ s.t.\overset{n}{\underset{j=1}{\sum }}\lambda_{j}X_{j}+s^{-}=\theta X_{j_{0}}\\ \overset{n}{\underset{j=1}{\sum }}\lambda_{j}Y_{j}-s_{j}=Y_{j_{0}}\\ \lambda \geq 0,\;\;s^{-}\geq 0,\;\;s_{j}\geq 0\left(j=1,2,\cdots ,n\right) \end{array}$$

Suppose the optimal solution of the CCR model is $\lambda^{*},\;\theta^{*},\;s^{-*},\;s_{j}^{*}\left(j=1,2,\cdots ,n\right)$. $\theta^{*}$ is the technical efficiency (TE) of each DMU. If $V_{D}<1$, then the DMU $j_{0}$ is non-DEA efficiency. The smaller the value is, the worse the efficiency will be. If $V_{D}=1$ and $s^{-*}=0,\;s_{j}^{*}\left(j=1,2,\cdots ,n\right)=0$*,* then the DMU $j_{0}$ is DEA efficiency. If $V_{D}=1$*,* $s^{-*}$and $s_{j}^{*}\left(j=1,2,\cdots ,n\right)$ are not all zero, then the DMU $j_{0}$ is relatively DEA efficient.

The BBC model is constructed as Equation (2):

(2) $$\begin{array}{c} min\delta -\varepsilon \left(s^{-}+\overset{m}{\underset{j=1}{\sum }}s_{j}\right)=V_{D}\\ s.t.\overset{n}{\underset{j=1}{\sum }}\lambda_{j}X_{j}+s^{-}=\theta X_{j_{0}}\\ \overset{n}{\underset{j=1}{\sum }}\lambda_{j}=1\\ \overset{n}{\underset{j=1}{\sum }}\lambda_{j}Y_{j}-s_{j}=Y_{j_{0}}\\ \lambda \geq 0,\;\;s^{-}\geq 0,\;\;s_{j}\geq 0\left(j=1,2,\cdots ,n\right) \end{array}$$

Assume that the optimal solution of the BCC model is $\delta^{*}$, which represents the pure technical efficiency (PTE) of each DMU. According to the TE obtained by the CCR model, the scale efficiency (SE) can be solved as *SE = TE/PTE*.

### 2.2. Indicator Selection and Data Description

In the choice of input indicators, the fiscal policy of environmental governance can serve as a representative indicator. The fiscal expenditure of environmental protection can directly reflect the fiscal expenditure policy, while the pollutant discharge fee can reflect the fiscal revenue policy of environmental pollution control. To describe the combined effect of the two policies, this paper selects the fiscal expenditure of environmental protection, which eliminates the pollutant discharge fee as the input indicator of the fiscal expenditure policy, and the pollutant discharge fee as the input indicator of the fiscal revenue policy. The input indicators are deflated by the GDP deflator with 2007 as the base year, and the unit is 10^8^ Chinese Yuan (CNY). For output indicators, this paper selects the environmental pollution treatment effects of waste- water, solid waste and waste gas. In the treatment of wastewater pollution, sanitary wastewater and industrial wastewater are selected as representatives. Sanitary wastewater treatment is expressed by the sewage treatment capacity of municipal sewage treatment plants, and the unit is 10^4^ cubic meters. Industrial wastewater treatment discharge is expressed by the discharge and treatment capacity of industrial wastewater, and the unit is 10^4^ tons. In solid waste pollution control, domestic garbage and industrial solid waste are selected as representatives. The treatment of domestic garbage is expressed by the amount of harmless disposal of urban household waste, and the unit is 10^4^ tons. The treatment of industrial solid waste is expressed by the amount of comprehensive utilization of industrial solid waste, and the unit is 10^4^ tons. In the treatment of waste gas pollution, the treatment effect of ${\mathrm{SO}}_{2}$ is selected as representative. Since the statistical aperture of ${\mathrm{SO}}_{2}$ is not uniform, the treatment effect of ${\mathrm{SO}}_{2}$ is expressed by the ratio of ${\mathrm{SO}}_{2}$ emissions/ 10^4^ CNY of GDP in the base period and ${\mathrm{SO}}_{2}$ emissions/ 10^4^ CNY of GDP in the current year. The larger the ratio is, the better the treatment effect of ${\mathrm{SO}}_{2}$ will be. The above indicators and instructions are shown in Table 1.

### 2.3. Research Data

Fiscal expenditure of environmental protection in China has been formally included in the fiscal budget since 2007. Based on the available data, this paper takes the data of 30 provincial-level administrative divisions from 2007 to 2017 in the fiscal policy of environmental governance of domestic and industrial pollution as an example for empirical analysis. Tibet, Hong Kong, Macao, and Taiwan are not included in the research object due to a lack of data. The data were collected from *Finance Yearbook of China* (2008–2018) [8], *China Statistical Yearbook* (2008–2018) [61], *China Statistical Yearbook on Environment* (2008–2018) [62] and *China Environment Yearbook* (2008–2018) [63].

To reflect socioeconomic development in different regions of China, 30 provinces are grouped into eastern, central, western and northeastern regions in this paper [64,65]. The eastern region contains 10 provinces and cities: Beijing, Shanghai, Tianjin, Hebei, Jiangsu, Zhejiang, Fujian, Shandong, Guangdong and Hainan. The central region contains 6 provinces: Shanxi, Anhui, Jiangxi, Henan, Hubei and Hunan. The western region contains 11 provinces and cities: Inner Mongolia, Guangxi, Chongqing, Sichuan, Guizhou, Yunnan, Shaanxi, Gansu, Qinghai, Ningxia and Xinjiang. The northeastern region contains 3 provinces, Liaoning, Jilin, and Heilongjiang. After the input and output indicators were selected, correlation analysis was conducted on the data from 2007 to 2017 as shown in Appendix A Table A2 [66]. The results indicate that the absolute value of correlation coefficients between the input and output indicators are greater than 0.6, meaning that there is a significant correlation between them. The correlation coefficients between input indicators and between output indicators are less than 0.6, indicating that there is no strong correlation between them. The correlation coefficients conform to the principle of indicator selection.

## 3. Efficiency Measurement and Convergence Analysis

### 3.1. Efficiency of Fiscal Policies for Environmental Pollution Control

Based on the input data of environmental fiscal policy and output data of environmental pollution control from 2007 to 2017 in 30 provincial-level administrative divisions, the TE, PTE and SE values of fiscal policy for environmental pollution control are calculated by using CCR and BCC models. The TE values of all divisions are shown in Table 2. In addition, the results of the PTE and SE values of fiscal policy for environmental pollution control are presented in Appendix A Table A3 and Table A4, respectively. Average values of the eastern region, the western region, the central region, the northeast region and the national are shaded to give a clearer picture. Similar treatments are given in the following paragraphs.

Table 2 shows that there is regional heterogeneity in the efficiency of fiscal policies for environmental pollution control in China. Most provinces in the eastern region are effective; that is, compared with the fiscal policy input of environmental pollution control in other provinces, their output reaches the optimal allocation. The efficiency of Guangxi in the western region also reached the optimal level.

**Figure 1:**
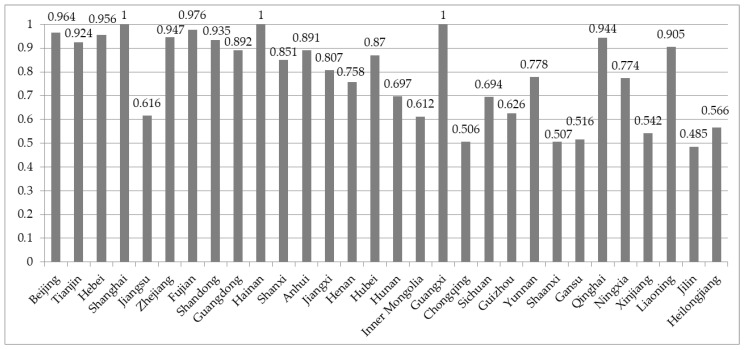
Mean values of TE in provincial-level administrative divisions, China (2007–2017).

In order to analyze the TE values of different divisions in China more intuitively, we draw a bar diagram of the mean values of TE as shown in Figure 1. It reveals that one-third of provinces in China exceed 0.9 in the mean values of TE. The mean technical efficiency of fiscal policies in Chongqing, Shaanxi, Gansu, Xinjiang, Jilin and Heilongjiang provinces is lower than 0.6, so there is considerable room for improvement in their efficiency. The efficiency of fiscal policies for environmental pollution control in all regions is ranked from high to low in the following order: eastern region, central region, western region and northeast region. 

**Figure 2:**
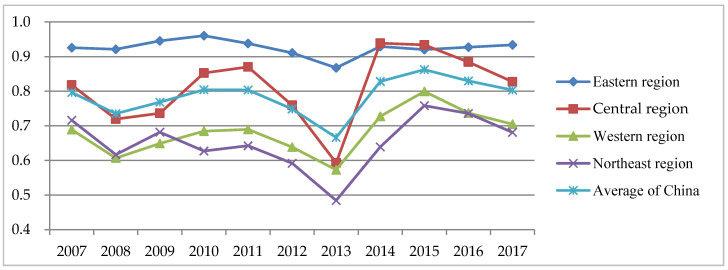
Change trend of TE values in each region of China (2007–2017).

To reflect the variation trend of fiscal policy efficiency in various regions more intuitively, a line chart of the TE values of fiscal policy for environmental pollution control in each region is shown in Figure 2. It shows that the variation trend of TE in different regions is basically consistent. From 2008 to 2010, the TE was generally on the rise. From 2011 to 2013, it was on the decline, and it reached its minimum in 2013. Since 2014, TE values had greatly improved, although they began to decline slightly in 2016. The possible reason for the declining TE values in 2013 was that local governments in China continue to increase financial investment in environmental pollution control, but the effect of environmental governance is not obvious. From 2013 to 2015, the TE values increased significantly, which was mainly due to the formulation of national policies, especially the proposal of ecological civilization construction in China. The government and sewage enterprises had attached great importance to environmental pollution control. Environmental regulation and other means of environmental protection also led to a substantial increasing in the TE values. Since 2016, the TE values had entered a bottleneck period. There were some rising spaces in the level of government performance management. 

From the perspective of regional differences, TE values of fiscal policy in the eastern region are obviously higher than those in other regions. Since 2014, the TE values of the central region and the eastern region have been similar. The gap of TE values between the western region and the northeastern region is small, but TE values in the western region are slightly higher than those in the northeastern region overall. It is obvious that developed regions have more efficient fiscal policies. The initial conclusion is that there is a certain relationship between the TE values of fiscal policies for environmental pollution control and the level of economic development.

**Figure 3:**
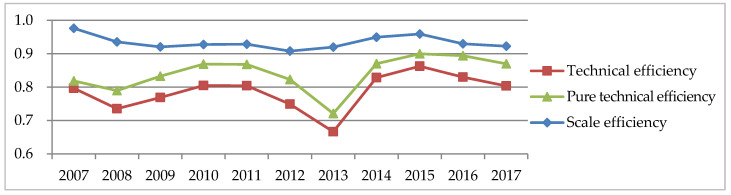
Change trend of TE (technical efficiency), PTE (pure technical efficiency) and SE (scale efficiency) values in China (2007–2017).

To analyze the relationship between TE, PTE and SE values, we draws a line diagram of the three ones, as shown in Figure 3. The fluctuating trend of TE values is similar to that of PTE values, which proves that changes in TE values are caused mainly by changes in PTE values. SE values are greater than 0.9 and less than 1, indicating that the ratio between the actual scale and the optimal scale is greater than 0.9; that is, there is a small gap between the actual scale and the maximum scale. When the efficiency of fiscal policies for environmental pollution control due to the level of system and management, namely, PTE, is taken into account without considering the SE, the overall level improves compared with the TE, and it reaches its maximum in 2015.

### 3.2. Malmquist Index and Decomposition Values

Based on the EFPE values in 30 provincial-level administrative divisions from 2007 to 2017, the growth rate of technical efficiency is calculated, that is, the Malmquist index. The Malmquist index is decomposed into efficiency change (EFFCH) and technical change (TECHCH), and EFFCH is decomposed into pure technical efficiency change (PECH) and scale efficiency change (SECH). The calculation results are shown in Table 3.

**Figure 4:**
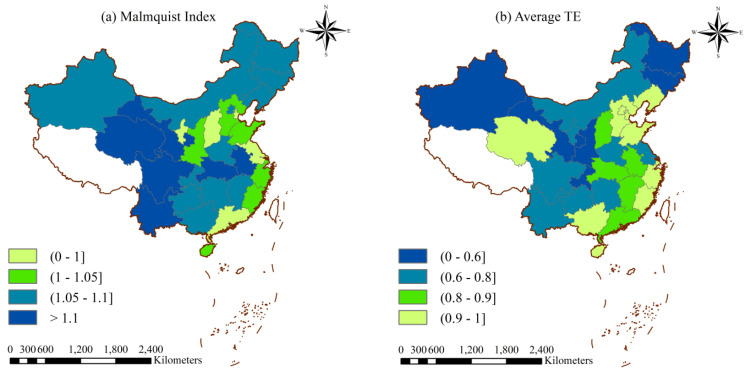
Horizontal interval distribution of (**a**) Malmquist index and (**b**) average TE in China (2007–2017).

As Table 3 shows, the Malmquist index values of Beijing, Shanghai, Fujian, Shandong, Guangdong, Hainan, Guangxi and Qinghai are affected only by technological progress. The technological progress in the efficiency of fiscal policy comes mainly from some intangible factors, such as policy orientation, market environment and organizational innovation. The horizontal interval distribution of the Malmquist index in China from 2007 to 2017 is presented in Figure 4a. From a regional perspective, the efficiency of fiscal policy grows faster in the western region, followed by the northeastern, central regions and the eastern region which exhibits a slight decline. The average TE is negatively correlated with the Malmquist index basically, as shown in Figure 4b, which means that the growth rate of the low efficient region is greater than that of the high efficient region. The policy efficiency of regions with low efficiency has greater room for improvement, so it is assumed that there is a regional convergence in the EFPE values.

### 3.3. Convergence Analysis

Absolute β convergence indicates that regions with similar cultures, institutions and other factors have the same TE status and regions with low TE values have a faster growth rate than those with high TE values. All regions will eventually tend to the same TE values in the end. In this paper, the absolute β convergence test method is adopted to investigate the convergence of EFPE values in various regions of China according to the TE values of fiscal policy for environmental pollution control calculated above. Based on the analysis of Barro and Sala-I-Martin (1992) [67], Equation (3) is used in this paper to test the convergence of regional TE values:

(3) $$\gamma_{it}=\alpha -\beta lnTE_{i0}+\varepsilon_{it}$$

where $\gamma_{it}$ represents the growth rate of the technical efficiency of fiscal policy in each region from period 0 to T, $TE_{i0}$ represents the technical efficiency of fiscal policy in period 0, $\varepsilon_{it}$ represents the random disturbance term, α represents the constant term and β represents the coefficient of $lnTE_{i0}$.

The above is a simplified test of the absolute convergence model. When the regression result β is positive, it shows that there is absolute convergence. Otherwise, there is no absolute convergence. EViews 8 was used to obtain the final convergence test results, as shown in Table 4.

According to the test results, the β coefficient of the whole country is positive, and the result is significant, indicating that the technical efficiency of fiscal policies for environmental pollution control has absolute convergence nationwide. The difference in the degree of technical efficiency is shrinking, which may be related to the mutual learning of policy experience among provinces. The β coefficients of the eastern, central and western regions are positive, and the results are significant, indicating that the differences in efficiency within different regions are also decreasing due to the positive externality of fiscal policies for environmental governance. The convergence speed varies from region to region, with the central region having a faster convergence speed. The reason may be that most provinces in the central region are located in the Yangtze River Economic Belt. The green development strategy “promoting well-coordinated environmental protection and avoiding excessive development” of the Yangtze River Economic Belt is conducive to narrowing the gap in the efficiency of fiscal policies for environmental pollution control in the central region.

## 4. The Spatial Effect of Fiscal Decentralization on EFPE

### 4.1. Index Selection and Data Description

- Explained variable: The technical efficiency (TE) of fiscal policies for environmental pollution control is the explained variable.
- Explanatory variables: To comprehensively measure the impact of fiscal decentralization on environmental fiscal efficiency, fiscal expenditure decentralization (FED) and fiscal revenue decentralization (FRD) are selected as representative indicators of fiscal decentralization [68]. The decentralization of local fiscal expenditure = per capita fiscal expenditure at the local level/(per capita fiscal expenditure at the local level + per capita fiscal expenditure at the central level). The decentralization of local fiscal revenue = per capita local fiscal revenue/(per capita local fiscal revenue + per capita central fiscal revenue).Control variables: To avoid endogeneity problems caused by the omission of variables, the following additional variables that may affect efficiency are selected as control variables. Economic development (ECON) [53] is measured by GDP per capita and deflated by the GDP deflator with 2007 as the base year, and the unit is CNY/person. Population density (POP) [57] is measured by the total population/district area at the end of the year, and the unit is people/${\mathrm{km}}^{2}$. The proportion of industry (IND) [53] is measured by the proportion of industrial added value in GDP, and the unit is %. Energy structure (ENER) [50] is measured by the proportion of coal consumption out of total energy consumption. The relevant proportion is calculated according to the raw coal conversion ratio of 0.7143, and the unit is %. Technical research (TECH) [57] is measured by the annual per capita R&D (research and development) expenditure on R&D personnel; that is, the internal expenditure on R&D divided by R&D personnel is equivalent to full-time equivalent positions. The GDP index based on 2007 is used for the reduction, and the unit is 10^4^ CNY/person.

This paper takes the data of TE, FED, FRD, ECON, POP, IND, ENER and TECH of 30 provincial-level administrative divisions in China from 2007 to 2017 as examples for empirical analysis. The data are compiled and calculated from *China Statistical Yearbook* (2008–2018) [61], *China Regional Economic Statistical Yearbook* (2008–2018) [69], *China Energy Statistical Yearbook* (2008–2018) [70] and *China High-tech Industry Statistical Yearbook* (2008–2018) [71]. The multicollinearity test of independent variable data shows that there is no multicollinearity among the variables.

### 4.2. Spatial Autocorrelation

The global Moran I index is usually used to test the spatial autocorrelation of variables and can be used to estimate the degree of spatial concentration. In Equation (4), $\varphi_{i}$ is the attribute value of element i, $\bar{\varphi }$ is the mean value of all attribute values, $\theta_{ij}$ is the spatial weight matrix, and $s^{2}$ is the variance value of φ. The variation range of the global Moran I index is [−1, 1], where Moran’s I > 0 represents the positive spatial autocorrelation, and the greater the value, the stronger the positive autocorrelation is. In contrast, Moran’s I < 0 represents the negative autocorrelation of space, and the smaller the value is, the stronger the negative autocorrelation is. Moran’s I = 0 means that space is random. After the global Moran I index is calculated, the standardized statistic z-value is often used to test the significance level of spatial autocorrelation:

(4) $$I=\frac{\sum_{i}^{n}\sum_{j\neq i}^{n}\theta_{ij}\left(\varphi_{i}-\bar{\varphi }\right)\left(\varphi_{j}-\bar{\varphi }\right)}{s^{2}\sum_{i}^{n}\sum_{j\neq i}^{n}\theta_{ij}}$$

In this paper, the binary adjacency matrix is used as the spatial weight matrix, and GeoDa is used to calculate and test the significance of the global Moran I index (*I* value) of the efficiency of fiscal policy for environmental pollution control in 30 provincial-level administrative divisions of China from 2007 to 2017. The results are shown in Table 5.

Table 5 shows that from 2007 to 2017, the global Moran I index of efficiency in China is positive and passes the significance test of 5%. Therefore, the efficiency of fiscal policies for environmental pollution control in 30 provincial-level administrative divisions of China presents a positive spatial autocorrelation. The results conform to the precondition of the spatial measurement model and verify the convergence of efficiency.

### 4.3. Spatial Econometric Model

The spatial econometric model is applicable when the explanatory variables, the explained variables and the control variables have spatial autocorrelation. The spatial lag model (SLM) is a spatial model representing the time-dependent relationship by introducing the lag term of dependent variables to reflect the direct interaction between dependent variables. The SLM is as Equation (5):

(5) Y=ρWy+Xβ+ε

where Y is the dependent variable, X is the independent variable, W is the spatial weight matrix, ρ is the spatial dependence degree of the dependent variable, ε is the disturbance term, and Wy is the spatial lag of the dependent variable.

According to the theoretical basis of the competition effect and demonstration effect of fiscal decentralization guiding local governments, as well as the test results of the spatial autocorrelation of the efficiency of fiscal policies for environmental pollution control, this paper uses SLM to analyze the spatial effect of fiscal decentralization on the efficiency of fiscal policies for environmental pollution control. The formula is as Equation (6):

(6) $$\begin{array}{cc} LnTE_{t}= & \rho WLnTE_{t-1}+\beta_{1}LnFED_{t}+\beta_{2}LnFRD_{t}+\beta_{3}LnECON_{t}\\ & +\beta_{4}LnPOP_{t}+\beta_{5}LnIND_{t}++\beta_{6}LnENER_{t}+\beta_{7}LnTECH_{t}+\varepsilon \end{array}$$

### 4.4. Spatial Effect Analysis

Based on the provincial panel data of 30 divisions in China from 2007 to 2017, the SLM was adopted to estimate the spatial effect of fiscal decentralization on the efficiency of fiscal policies for environmental pollution control. The results are shown in Table 6.

The results show that fiscal expenditure decentralization, economic development and industrial proportion have significant direct and indirect effects (spatial spillover effects) on EFPE. The spatial effect of fiscal revenue decentralization, population density, energy structure and technology research are not significant.

Decomposing the spatial effect, it demonstrates that fiscal expenditure decentralization has a significant direct negative effect, which indicates that the improvement of fiscal expenditure decentralization will reduce EFPE. This improvement occurs because when local governments have more autonomy in fiscal expenditure, they will prioritize economic development under the current government assessment system. Fiscal expenditure decentralization also has a significant negative spatial spillover effect on EFPE, indicating that the improvement of fiscal expenditure decentralization in a local division will have a negative impact on the EFPE of its neighboring divisions. The possible reason is that there are competition effects among neighboring areas, resulting in economic growth comparison that is detrimental to EFPE indirectly. As fiscal revenue is affected by transfer payment from the central government, the change is more stable in the longer term, which results in fiscal revenue decentralization having no obvious influence on the decision-making behavior of the local governments in fiscal policies for environmental governance. Therefore, fiscal revenue decentralization has no significant influence on EFPE.

Economic development has a significant direct positive effect on EFPE because economically developed regions have less urgency to achieve economic development instead of pursuing high-quality development. Meanwhile, economically developed regions have mature experience in government work and better management standards. These findings verified the previous inference of the relationship between EFPE and the level of economic development. Economic development also has a significant positive spatial spillover effect on EFPE, which indicates that the improvement of the economic development level in a local division will have a positive impact on the EFPE of its neighboring divisions. Industrial proportion has a significant direct negative effect on EFPE because division with a higher proportion of industry is confronted with greater pressure in environmental governance, and the legacy of environmental pollution is even greater. The ratio of industrial occupancy also has a significant negative spatial spillover effect on EFPE, indicating that the ratio of industrial occupancy in a local division has a negative effect on the EFPE of its neighboring divisions. A possible reason is that environmental pollution has negative externality, which leads to a reduction of EFPE in neighboring divisions.

## 5. Conclusions and Suggestions

This paper is devoted to measure the efficiency of fiscal policies for environmental pollution control, discuss the spatial effect of fiscal decentralization and the control variables, and propose corresponding policy suggestions that have important theoretical value and practical significance for China. By calculating the efficiency of fiscal policies for environmental pollution control in 30 provincial-level administrative divisions of China from 2007 to 2017 and conducting an empirical analysis of the spatial effect of fiscal expenditure decentralization and fiscal revenue decentralization, we obtain the following conclusions:

- EFPE has been improved greatly overall since 2014. Although it declined slightly in 2016 and 2017, the level is still high. The change in TE is caused mainly by the change in PTE, while SE is not the main reason for the change in TE. There is regional heterogeneity in EFPE of China. EFPE values in the eastern region are significantly higher than those in other regions. The growth rate of the low efficient region is greater than that of the high efficient region. There is a regional convergence in the EFPE values, and the central region has a relatively fast convergence rate.
- Fiscal expenditure decentralization has a significant direct negative effect and spatial spillover effect on EFPE. Fiscal revenue decentralization has a non-significant impact on EFPE due to fiscal transfer payment. Economic development will still be an important consideration for local governments under current government assessment, and neighboring areas are more likely to compare the economic growth. Economic development has a positive spatial effect and industrial proportion has a negative spatial effect on EFPE.

To improve the efficiency of fiscal policies for environmental pollution control and environmental quality in China, this paper proposes three aspects of policy suggestions as follows:

- The level of fiscal input of environmental governance should be increased, and the central fiscal transfer payment system for environmental protection should be improved. On the basis of the current control of water, soil and air pollution, we should further refine fiscal policies for different pollution types. For example, during the implementation of domestic garbage classification in China, financial support for domestic garbage classification should be increased. We can improve the regulatory role of the central government’s transfer payment policy in environmental governance. Fiscal expenditure for areas with high environmental pressure, heavy tasks and financial pressure increase should be supported to balance the relationship between economic development and environmental protection.
- The government performance assessment system should be reformed, and the conditions of government expenditure on environmental pollution control can be innovated. Government performance assessments should consider not only the scale but also the efficiency of fiscal expenditure on environmental pollution control. The central government may explore setting regional and periodical performance assessment targets for fiscal budgets of environmental governance. Local governments may carry out a whole-process performance evaluation of the pollution control project, and the evaluation subject can be the local government or the third-party institution. By changing the condition of fiscal expenditure, the policy of replacing compensation with reward can be implemented based on the results of pollution control.
- Horizontal fiscal cooperation in cross-regional governance can be promoted to realize regionally coordinated governance. Water pollution, air pollution and other environmental pollution problems have spillover effects among divisions. Therefore, coordination and integration of the fiscal policies should be implemented based on the principle of matching administrative and financial powers in environmental pollution control. By breaking the boundaries of administrative divisions and exploring the horizontal allocation of environmental funds, a cross-domain model of environmental pollution governance can be established. A fiscal expenditure mechanism for environmental protection should be established, so the coordinated regional development can be promoted in China.

## Figures and Tables

**Table 1:** Indicators of input and output in the efficiency measurement of fiscal policies for environmental pollution control.

Variable	Classification	Indicator	Definition of Indicator
Inputs	Fiscal expenditure policy	Fiscal expenditure of environmental protection-pollutant discharge fee	Environmental fiscal expenditure excluding pollutant discharge fee
	Fiscal revenue policy	Pollutant discharge fee	Pollutant discharge fee collected by local governments
Outputs	Pollution treatment of wastewater	Sanitary wastewater treatment	Sewage treatment capacity of municipal sewage treatment plants [34]
		Industrial wastewater treatment	Discharge and treatment capacity of industrial wastewater
	Pollution treatment of solid waste	Domestic garbage treatment	The amount of harmless disposal of urban household waste [34]
		Industrial solid waste treatment	Amount of comprehensive utilization of industrial solid waste [33]
	Pollution treatment of waste gas	Treatment effect of ${\mathrm{SO}}_{2}$	Ratio of ${\mathrm{SO}}_{2}$ emissions/ 10^4^ CNY of GDP in the base period and ${\mathrm{SO}}_{2}$ emissions/10^4^ CNY of GDP in the current year

Note: CNY = Chinese Yuan; GDP = gross domestic product.

**Table 2:** Technical efficiency (TE) values of fiscal policies for environmental pollution control in China (2007–2017).

Division	2007	2008	2009	2010	2011	2012	2013	2014	2015	2016	2017
Beijing	1	1	1	1	1	1	1	0.857	0.751	1	1
Tianjin	1	1	1	1	1	0.965	0.796	0.802	0.931	1	0.674
Hebei	1	0.936	0.91	1	1	1	0.995	1	0.881	0.897	0.892
Shanghai	1	1	1	1	1	1	1	1	1	1	1
Jiangsu	0.632	0.541	0.588	0.885	0.619	0.613	0.606	0.676	0.654	0.684	0.775
Zhejiang	0.626	0.791	1	1	1	1	1	1	1	1	1
Fujian	1	1	1	1	1	1	0.984	1	0.992	0.756	1
Shandong	1	0.945	1	1	1	0.842	0.564	1	0.995	0.935	1
Guangdong	1	1	0.957	0.72	0.76	0.687	0.732	0.959	1	1	1
Hainan	1	1	1	1	1	1	1	1	1	1	1
Eastern region	0.926	0.921	0.946	0.961	0.938	0.911	0.868	0.929	0.920	0.927	0.934
Shanxi	0.629	0.603	0.792	1	1	1	0.454	1	1	1	0.878
Anhui	1	0.986	0.984	0.842	0.879	0.748	0.553	0.985	1	1	0.822
Jiangxi	0.817	0.653	0.672	0.846	1	0.703	0.632	1	0.919	0.866	0.768
Henan	0.719	0.652	0.665	0.772	0.833	0.724	0.612	0.925	0.885	0.797	0.752
Hubei	0.94	0.822	0.748	0.995	0.831	0.768	0.696	1	0.928	0.843	1
Hunan	0.802	0.602	0.557	0.661	0.678	0.613	0.611	0.724	0.872	0.801	0.744
Central region	0.818	0.720	0.736	0.853	0.870	0.759	0.593	0.939	0.934	0.885	0.827
Inner Mongolia	0.945	0.522	0.534	0.567	0.689	0.486	0.301	0.619	0.654	0.71	0.709
Guangxi	1	1	1	1	1	1	1	1	1	1	1
Chongqing	0.396	0.347	0.524	0.526	0.47	0.394	0.437	0.591	0.591	0.688	0.605
Sichuan	0.853	0.817	0.589	0.682	0.693	0.561	0.457	0.637	0.839	0.716	0.787
Guizhou	0.487	0.473	0.488	0.631	0.708	0.728	0.74	0.753	0.841	0.5	0.538
Yunnan	0.91	0.873	0.937	0.9	0.689	0.596	0.495	0.738	1	0.727	0.696
Shaanxi	0.511	0.403	0.449	0.493	0.536	0.479	0.4	0.58	0.653	0.663	0.406
Gansu	0.501	0.357	0.384	0.49	0.34	0.435	0.37	0.513	0.52	0.938	0.829
Qinghai	1	0.985	0.829	0.838	1	1	0.73	1	1	1	1
Ningxia	0.532	0.533	0.892	0.903	0.843	0.886	0.965	1	1	0.528	0.433
Xinjiang	0.445	0.362	0.512	0.501	0.62	0.46	0.405	0.57	0.696	0.639	0.749
Western region	0.689	0.607	0.649	0.685	0.690	0.639	0.573	0.727	0.799	0.737	0.705
Liaoning	0.648	0.729	0.889	0.874	1	1	0.819	1	1	1	1
Jilin	0.673	0.433	0.492	0.479	0.391	0.355	0.32	0.39	0.671	0.556	0.574
Heilongjiang	0.827	0.688	0.664	0.529	0.537	0.421	0.314	0.527	0.604	0.651	0.469
Northeastern region	0.716	0.617	0.682	0.627	0.643	0.592	0.484	0.639	0.758	0.736	0.681
Average of China	0.796	0.735	0.769	0.804	0.804	0.749	0.666	0.828	0.863	0.830	0.803

**Table 3:** Malmquist index and decomposition values of EFPE in China (2007–2017).

	Index	EFFCH	PECH	SECH	TECHCH	Malmquist Index
Division						
Beijing		1	1	1	1.071	1.071
Tianjin		0.961	0.964	0.998	0.988	0.949
Hebei		0.989	1	0.989	1.061	1.049
Shanghai		1	1	1	1.014	1.014
Jiangsu		1.021	1	1.021	0.957	0.977
Zhejiang		1.048	1.048	1	0.955	1.001
Fujian		1	1	1	1.011	1.011
Shandong		1	1	1	1.016	1.016
Guangdong		1	1	1	0.974	0.974
Hainan		1	1	1	1.049	1.049
Eastern region		1.002	1.001	1.001	1.010	1.011
Shanxi		1.034	1.029	1.005	0.945	0.977
Anhui		0.981	1	0.981	1.141	1.119
Jiangxi		0.994	1.02	0.974	1.059	1.052
Henan		1.005	1.005	1	1.069	1.073
Hubei		1.006	1.004	1.002	1.121	1.128
Hunan		0.993	0.999	0.994	1.07	1.062
Central region		1.002	1.01	0.993	1.068	1.069
Inner Mongolia		0.972	0.979	0.993	1.091	1.06
Guangxi		1	1	1	1.07	1.07
Chongqing		1.043	1.051	0.993	1.045	1.091
Sichuan		0.992	0.985	1.007	1.11	1.101
Guizhou		1.01	1.012	0.998	1.088	1.099
Yunnan		0.974	0.987	0.987	1.196	1.165
Shaanxi		0.977	0.987	0.991	1.071	1.047
Gansu		1.052	1.062	0.99	1.159	1.218
Qinghai		1	1	1	1.129	1.129
Ningxia		0.98	1.034	0.947	1.003	0.982
Xinjiang		1.053	1.084	0.971	1.029	1.084
Western region		1.005	1.016	0.989	1.090	1.095
Liaoning		1.044	1.044	1	1.034	1.08
Jilin		0.984	0.999	0.985	1.077	1.06
Heilongjiang		0.945	0.945	1	1.145	1.081
Northeast region		0.991	0.996	0.995	1.085	1.074
Average of China		1.002	1.008	0.994	1.056	1.058

Note: EFPE = the efficiency of fiscal policies for environmental pollution control; EFFCH = efficiency change; SECH = scale efficiency change; PECH = pure technical efficiency change; TECHCH = technical change.

**Table 4:** Convergence test results of EFPE values in regions of China (2007–2017).

	Region	Whole Country	Eastern Region	Central Region	Western Region	Northeastern Region
Coefficient						
α		0.99	0.99	0.99	0.99	0.90
*p* value of α		0	0	0	0	0.04
β		0.08	0.09	0.13	0.08	0.32
*p* value of β		0	0.02	0.01	0.01	0.31
*R* squared adjusted		0.53	0.44	0.86	0.52	0.56

**Table 5:** Spatial autocorrelation indexes of EFPE values in China (2007–2017).

Index	2007	2008	2009	2010	2011	2012	2013	2014	2015	2016	2017
*I* value	0.118	0.277	0.28	0.324	0.213	0.172	0.284	0.234	0.127	0.13	0.202
*Z* value	1.663	2.59	2.617	3.025	2.15	1.704	2.696	2.324	1.784	1.854	1.941
*p* value	0.03	0.009	0.005	0.002	0.028	0.041	0.014	0.017	0.043	0.035	0.033

**Table 6:** Direct effect, indirect effect and total effect of fiscal decentralization on EFPE in China (2007–2017).

	Variable	Direct Effect	Indirect Effect	Total Effect
Explanatory variables	FED	−1.547 ** (−2.16)	−1.079 * (−1.84)	−2.625 ** (−2.09)
	FRD	−0.060 (−0.26)	−0.042 (−0.26)	−0.102 (−0.26)
Control variables	ECON	0.513 ** (2.53)	0.358 ** (2.05)	0.871 ** (2.42)
	POP	−0.379 (−1.21)	−0.264 (−1.15)	−0.643 (−1.20)
	IND	−0.541 *** (−3.11)	−0.377 ** (−2.48)	−0.918 *** (−3.02)
	ENER	0.105 (0.97)	0.073 (0.91)	0.178 (0.95)
	TECH	0.013 (0.36)	0.009 (0.36)	0.023 (0.36)

Note: *, ** and *** represents significance levels of 10%, 5% and 1%, respectively. The value in parentheses is the z-statistic.

## Appendix A

**Table A1:** The amount (10^8^ CNY) and proportion (%) of the fiscal expenditure on environmental protection in China (2007–2017).

	Total Amount in Whole Country	Central Government Expenditure		Local Government Expenditure		Proportion of Fiscal Expenditure	Proportion of GDP
		Total Amount	Proportion of Whole Country	Total Amount	Proportion of Whole Country		
2007	995.82	34.59	3.47%	961.24	96.53%	1.94%	0.37%
2008	1451.36	66.21	4.56%	1385.15	95.44%	2.37%	0.45%
2009	1934.04	37.91	1.96%	1896.13	98.04%	2.82%	0.55%
2010	2441.98	69.48	2.85%	2372.50	97.15%	2.94%	0.59%
2011	2640.98	74.19	2.81%	2566.79	97.19%	2.54%	0.54%
2012	2963.46	63.65	2.15%	2899.81	97.85%	2.53%	0.55%
2013	3435.15	100.26	2.92%	3334.89	97.08%	2.66%	0.58%
2014	3815.64	344.74	9.03%	3470.90	90.97%	2.72%	0.59%
2015	4802.89	400.41	8.34%	4402.48	91.66%	3.15%	0.70%
2016	4734.82	295.49	6.24%	4439.33	93.76%	2.52%	0.65%
2017	5617.33	350.56	6.24%	5266.77	93.76%	2.76%	0.72%

Note: Calculation based on data from *Finance Yearbook of China* (2008–2018) [8].

**Table A2:** Correlation coefficients of input and output indicators in the efficiency measurement of fiscal policies for environmental pollution control.

Indicators	$O_{1}\;$	$O_{2}\;$	$O_{3}\;$	$O_{4}\;$	$O_{5}\;$	$I_{1}\;$	$I_{2}\;$
$O_{1}$	1	0.410	0.349	0.292	0.576	0.798	−0.739
$O_{2}$	0.410	1	0.287	0.488	0.522	0.789	−0.672
$O_{3}$	0.349	0.287	1	0.296	0.278	0.173	−0.235
$O_{4}$	0.292	0.488	0.296	1	0.788	0.829	−0.747
$O_{5}$	0.576	0.522	0.278	0.788	1	0.761	−0.808
$I_{1}$	0.798	0.789	0.173	0.829	0.761	1	−0.316
$I_{2}$	−0.739	−0.672	−0.235	−0.747	−0.808	−0.316	1

Note:$\;O_{1}$ = sanitary wastewater treatment; $O_{2}$ = industrial wastewater treatment; $O_{3}$ = domestic garbage treatment; $O_{4}$ = industrial solid waste treatment; $O_{5}$ = treatment effect of ${\mathrm{SO}}_{2}$; $I_{1}$ = fiscal expenditure of environmental protection-pollutant discharge fee; $I_{2}$ = pollutant discharge fee.

**Table A3:** Pure technical efficiency values of fiscal policies for environmental pollution control in China (2007–2017).

Division	2007	2008	2009	2010	2011	2012	2013	2014	2015	2016	2017
Beijing	1	1	1	1	1	1	1	0.972	0.897	1	1
Tianjin	1	1	1	1	1	0.966	0.808	0.812	0.939	1	0.69
Hebei	1	1	1	1	1	1	1	1	1	1	1
Shanghai	1	1	1	1	1	1	1	1	1	1	1
Jiangsu	1	1	1	1	0.965	0.908	0.896	1	1	1	1
Zhejiang	0.626	0.86	1	1	1	1	1	1	1	1	1
Fujian	1	1	1	1	1	1	1	1	0.996	0.772	1
Shandong	1	1	1	1	1	1	0.711	1	1	1	1
Guangdong	1	1	1	1	1	1	1	1	1	1	1
Hainan	1	1	1	1	1	1	1	1	1	1	1
Eastern region	0.963	0.986	1.000	1.000	0.997	0.987	0.942	0.978	0.983	0.977	0.969
Shanxi	0.714	0.873	0.804	1	1	1	0.526	1	1	1	0.952
Anhui	1	1	1	0.984	1	1	0.562	1	1	1	1
Jiangxi	0.817	0.666	0.674	0.86	1	0.753	0.718	1	0.949	0.98	1
Henan	0.802	0.736	0.851	0.885	0.848	0.827	0.636	1	0.885	0.817	0.841
Hubei	0.962	0.847	0.894	1	1	0.906	0.784	1	0.942	0.862	1
Hunan	0.802	0.646	0.622	0.779	0.744	0.66	0.692	0.769	1	1	0.792
Central region	0.850	0.795	0.808	0.918	0.932	0.858	0.653	0.962	0.963	0.943	0.931
Inner Mongolia	0.972	0.64	0.651	0.682	1	0.606	0.337	0.763	0.802	0.836	0.786
Guangxi	1	1	1	1	1	1	1	1	1	1	1
Chongqing	0.396	0.348	0.578	0.566	0.49	0.493	0.514	0.673	0.622	0.696	0.649
Sichuan	0.923	1	0.757	0.83	0.826	0.728	0.502	0.766	0.839	0.731	0.796
Guizhou	0.487	0.528	0.533	0.663	0.727	0.745	0.759	0.814	0.871	0.53	0.55
Yunnan	0.91	0.898	1	0.923	1	0.852	0.513	0.801	1	0.88	0.794
Shaanxi	0.511	0.416	0.552	0.993	0.544	0.48	0.405	0.597	0.653	0.671	0.447
Gansu	0.501	0.37	0.412	0.512	0.391	0.462	0.39	0.53	0.54	0.945	0.914
Qinghai	1	1	0.909	0.845	1	1	0.806	1	1	1	1
Ningxia	0.532	0.538	1	1	0.897	0.914	0.985	1	1	1	0.743
Xinjiang	0.445	0.374	0.558	0.511	0.654	0.576	0.543	0.654	0.764	0.805	1
Western region	0.698	0.647	0.723	0.775	0.775	0.714	0.614	0.782	0.826	0.827	0.789
Liaoning	0.648	0.809	0.999	1	1	1	0.834	1	1	1	1
Jilin	0.673	0.435	0.505	0.501	0.407	0.372	0.336	0.398	0.672	0.609	0.666
Heilongjiang	0.832	0.692	0.672	0.53	0.543	0.43	0.363	0.546	0.614	0.683	0.473
Northeastern region	0.718	0.645	0.725	0.677	0.650	0.601	0.511	0.648	0.762	0.764	0.713
Average of China	0.818	0.789	0.832	0.869	0.868	0.823	0.721	0.870	0.900	0.894	0.870

**Table A4:** Scale efficiency values of fiscal policies for environmental pollution control in China (2007–2017).

Division	2007	2008	2009	2010	2011	2012	2013	2014	2015	2016	2017
Beijing	1	1	1	1	1	1	1	0.882	0.837	1	1
Tianjin	1	1	1	1	1	0.999	0.985	0.988	0.991	1	0.977
Hebei	1	0.936	0.91	1	1	1	0.995	1	0.881	0.897	0.892
Shanghai	1	1	1	1	1	1	1	1	1	1	1
Jiangsu	0.632	0.541	0.588	0.885	0.641	0.675	0.676	0.676	0.654	0.684	0.775
Zhejiang	1	0.920	1	1	1	1	1	1	1	1	1
Fujian	1	1	1	1	1	1	0.984	1	0.996	0.979	1
Shandong	1	0.945	1	1	1	0.842	0.793	1	0.995	0.935	1
Guangdong	1	1	0.957	0.72	0.76	0.687	0.732	0.959	1	1	1
Hainan	1	1	1	1	1	1	1	1	1	1	1
Eastern region	0.963	0.934	0.946	0.961	0.940	0.920	0.917	0.950	0.935	0.950	0.964
Shanxi	0.881	0.691	0.985	1	1	1	0.863	1	1	1	0.922
Anhui	1	0.986	0.984	0.856	0.879	0.748	0.984	0.985	1	1	0.822
Jiangxi	1	0.980	0.997	0.984	1	0.934	0.880	1	0.968	0.884	0.768
Henan	0.897	0.886	0.781	0.872	0.982	0.875	0.962	0.925	1	0.976	0.894
Hubei	0.977	0.970	0.837	0.995	0.831	0.848	0.888	1	0.985	0.978	1
Hunan	1	0.932	0.895	0.849	0.911	0.929	0.883	0.941	0.872	0.801	0.939
Central region	0.959	0.908	0.913	0.926	0.934	0.889	0.910	0.975	0.971	0.940	0.891
Inner Mongolia	0.972	0.816	0.820	0.831	0.689	0.802	0.893	0.811	0.815	0.849	0.902
Guangxi	1	1	1	1	1	1	1	1	1	1	1
Chongqing	1	0.997	0.907	0.929	0.959	0.799	0.850	0.878	0.950	0.989	0.932
Sichuan	0.924	0.817	0.778	0.822	0.839	0.771	0.910	0.832	1	0.979	0.989
Guizhou	1	0.896	0.916	0.952	0.974	0.977	0.975	0.925	0.966	0.943	0.978
Yunnan	1	0.972	0.937	0.975	0.689	0.700	0.965	0.921	1	0.826	0.877
Shaanxi	1	0.969	0.813	0.496	0.985	0.998	0.988	0.972	1	0.988	0.908
Gansu	1	0.965	0.932	0.957	0.870	0.942	0.949	0.968	0.963	0.993	0.907
Qinghai	1	0.985	0.912	0.992	1	1	0.906	1	1	1	1
Ningxia	1	0.991	0.892	0.903	0.940	0.969	0.980	1	1	0.528	0.583
Xinjiang	1	0.968	0.918	0.980	0.948	0.799	0.746	0.872	0.911	0.794	0.749
Western region	0.991	0.943	0.893	0.894	0.899	0.887	0.924	0.925	0.964	0.899	0.893
Liaoning	1	0.901	0.890	0.874	1	1	0.982	1	1	1	1
Jilin	1	0.995	0.974	0.956	0.961	0.954	0.952	0.980	0.999	0.913	0.862
Heilongjiang	0.994	0.994	0.988	0.998	0.989	0.979	0.865	0.965	0.984	0.953	0.992
Northeastern region	0.998	0.964	0.951	0.943	0.983	0.978	0.933	0.982	0.994	0.955	0.951
Average of China	0.976	0.935	0.920	0.928	0.928	0.908	0.920	0.949	0.959	0.930	0.922
